# Single-step Optimization in Triaging Large Vessel Occlusion Strokes: Identifying Factors to Improve Door-to-groin Time for Endovascular Therapy

**DOI:** 10.5811/westjem.59770

**Published:** 2023-06-30

**Authors:** Joshua Rawson, Ashley Petrone, Amelia Adcock

**Affiliations:** * West Virginia University School of Medicine, Morgantown, West Virginia; † West Virginia University, Department of Pathology, Morgantown, West Virginia; ‡ West Virginia University, Department of Neurology; Cerebrovascular Division, Morgantown, West Virginia

## Abstract

**Introduction:**

Although acute stroke endovascular therapy (EVT) has dramatically improved outcomes in acute ischemic stroke (AIS) patients with large vessel occlusions (LVO), access to EVT-capable centers remains limited, particularly in rural areas. Therefore, it is essential to optimize triage systems for EVT-eligible patients. One strategy may be the use of a telestroke network that typically consists of multiple spoke sites that receive a consultation to determine appropriateness of patient transfer to an EVT-capable hub site. Standardization of AIS protocols may be necessary to achieve target door-to-groin (DTG) times of less than 60 minutes in EVT-eligible patients upon hub arrival. Specifically, the decision to obtain vascular imaging at the transferring hub site vs delaying until arrival at the hub is controversial. The purpose of this study was to identify factors associated with reduced DTG time in LVO-AIS patients.

**Methods:**

We performed a retrospective chart review for all patients treated over a 3.5-year period at our home hub institution. Patients were classified as telestroke transfers, non-telestroke transfers, and direct-to-hub presentations. We recorded demographic information, DTG time, reperfusion status, length of stay (LOS), functional status at discharge, seven-day mortality, and the site where vascular imaging— computed tomography angiography (CTA)—was obtained. We performed binary logistic regression to identify factors associated with DTG <60 minutes.

**Results:**

In the sample of EVT-eligible patients (n = 383), CTA was performed at the spoke site prior to transfer to the hub institution in 53% of cases. Further, 59% of telestroke transfer cases received a CTA prior to transfer compared to only 40% of non-telestroke transfers (59 vs 40%, *P* = 0.01). A Door-to-groin time <60 minutes was achieved in 67% of transfer patients who received pre-transfer CTA compared to only 22% of transfer patients who received CTA upon hub arrival and 17% of patients who presented directly to the hub. Ultimately, transfer patients who received CTA prior to transfer were 7.2 times more likely to have a DTG <60 minutes compared to those who did not (OR 7.2, 95% confidence interval 3.5–14.7; *P* < 0.001).

**Conclusion:**

Pre-transfer computed tomography angiography was the only significant predictor of achieving target door-to-groin times of less than 60 minutes. Because DTG time has been well established as a predictor of clinical outcomes, including pre-transfer CTA in a standardized acute ischemic stroke protocol may prove beneficial. Our findings also illustrate the need to optimize direct-to-hub stroke alerts and telestroke relationships to minimize workflow disruptions, which became more apparent during the pandemic.

## INTRODUCTION

Endovascular therapy (EVT) for large vessel occlusion acute ischemic stroke (LVO-AIS) is now the standard of care for eligible patients.^1^ However, roughly half of all Americans, predominantly those residing in rural areas, lack timely (<60 minutes [min]) access to centers capable of performing EVT.^2^ Therefore, optimal triage systems for LVO-AIS patients are essential for emergency departments (ED) and health systems in rural regions that may be located hours from an EVT-capable center. Telestroke networks are a potent tool that improves clinical outcomes in resource-limited rural areas.^3^,^4^

Door-to-groin (DTG) time has been shown to be an independent predictor of favorable outcomes in AIS patients undergoing EVT.^5^,^6^ However, it remains unclear whether spoke-site participation in a hub’s telestroke network directly contributes to shorter DTG times. The timing of the vascular imaging required prior to a patient undergoing EVT must also be considered with respect to DTG time. The results of previous studies evaluating the effect of pre-transfer vascular imaging on DTG time have been inconsistent, with some studies indicating either prolonged or improved DTG.^7^–^9^ Therefore, it remains controversial as to whether computed tomography angiography (CTA) should be performed prior to transfer in patients with suspected LVO-AIS.

The COVID-19 pandemic highlighted inefficiencies in the triaging of AIS patients, particularly due to resource limitations and staffing issues. Although a large, single-center study detailed a decline of nearly 32% in stroke code activations at the beginning of the pandemic in April 2020, the rate of EVT remained consistent compared to the last several years.^10^ As COVID-19 continues to require the rapid reallocation of resources and staff, with further long-term effects on staffing, it is critical that EDs and stroke teams efficiently triage patients who may be candidates for EVT. Decreasing the number of futile transfers is also a key component of reducing the burden on hub centers. We previously reported an averted transfer rate of 65% using our telestroke network in West Virginia,^11^ a state in which 34 of its 55 counties are designated as either rural or super-rural. Consideration of factors that impact the triage process, such as pre-transfer CTA or access to telestroke consultation, serves to maximize the efficiency with which hub EDs and comprehensive stroke centers can provide care.

In this study, we sought to determine the primary factors contributing to target hub-DTG times in LVO-AIS patients undergoing EVT. Our overarching goal was to identify factors that may impact the efficiency of the triage process for stroke patients with LVOs in EDs and stroke centers.

## METHODS

We conducted this study at West Virginia University Hospital, a tertiary-care facility with a comprehensive stroke center that serves as the telestroke hub of 29 spoke hospitals. This organizational framework was previously described in more detail.^11^ This study received institutional review board approval, and the requirement for patient-informed consent requirement was waived based on retrospective chart review. Between January 2016–September 2021, we performed a retrospective chart review of all LVO-AIS patients who received EVT at our hub institution. This included direct-to-hub ED presentations, as well as transfer patients from spoke sites.

**Population Health Research Capsule**

What do we already know about this issue?

*Endovascular therapy (EVT) is a time-sensitive treatment for acute ischemic stroke (AIS) that has dramatically improved patient outcomes. It can only be performed at EVT-capable institutions*.

What was the research question?

*We sought* to *identify factors associated with reduced door-to-groin (DTG) times in AIS patients with large vessel occlusion*.

What was the major finding of the study?

*Transfer patients who received computed tomography angiography prior to transfer were 7.2 times more likely to have a DTG <60 min compared to those who did not*.

How does this improve population health?

*Triage systems must be optimized to provide timely EVT. This is of particular importance to patients in resource-limited rural areas*.

The hub-and-spoke model arranges service-delivery assets into a network consisting of an anchor establishment (hub), which offers a full array of services, complemented by secondary establishments (spokes), which offer more limited-service arrays, routing patients needing more intensive services to the hub for treatment. Our network had 29 spokes ranging from critical access hospitals to large community-academic centers across four states (West Virginia, Maryland, Ohio, and Pennsylvania). Furthermore, transfers were categorized as telestroke vs non-telestroke transfers.

Variables collected from each patient’s chart included the following: site of initial presentation (direct-to-hub vs transfer); telestroke spoke-site transfer; or non-telestroke site transfer. Other variables collected included National Institutes of Health Stroke Scale (NIHSS) scores upon arrival and at discharge, recanalization status (thrombolysis in cerebral infarction) after EVT, and hub length of stay. Functional outcome was assessed using the modified Rankin scale (mRS) at or within 90 days of discharge; patients with mRS >2 were considered to have poor functional outcome. Mortality rate within 90 days of discharge was also determined. We calculated DTG time for all patients, defined as the elapsed time between hub arrival time to groin puncture at the start of EVT.

We performed statistical analysis using SPSS Statistics 27.0 (IBM Corporation, Armonk, NY). All study variables were assessed for normality using Shapiro-Wilk testing to determine appropriate parametric or non-parametric tests for unadjusted comparisons between independent variables. We compared frequencies and proportions of study variables using a chi-square (x2) test. We used binary logistic regression to determine whether there was an association between study variables, including NIHSS score, telestroke vs non-telestroke, or pre-transfer CTA with a DTG <60 min. We verified that the data did not violate the assumptions of logistic regression, including performing a Box-Tidwell test to check for linearity between the predictors and the logit. We performed a backward stepwise regression model whereby each variable was entered in the first step of the model, and variables that remained in the last step of the model were considered significant predictors of DTG <60 min.

## RESULTS

A total of 383 patients met inclusion criteria for this study; 189 (49%) presented directly to our hub institution (direct-to-hub); and 194 (51%) arrived as a transfer. Median DTG time was significantly shorter in transfer patients compared to direct-to-hub patients (62 vs 83 min, *P* < 0.001). Similarly, DTG <60 min was achieved in a significantly higher proportion of transfer patients compared to direct-to-hub (47 vs 17%, *P* < 0.001).

Of the transfers, 71% received a telestroke consult prior to transfer. Median DTG time was significantly shorter in telestroke transfer to direct-to-hub patients (61 vs 83 min, *P* < 0.0001), and DTG <60 min was achieved in a significantly higher proportion of telestroke transfer patients compared to direct-to-hub (50 vs 17%, *P* < 0.001) (Table 1). There was no difference in median DTG time in telestroke compared to non-telestroke transfers (61 vs 78 min, *P* = 0.30), nor was there a difference in DTG <60 min in telestroke compared to non-telestroke transfers (50 vs 38%, *P* = 0.13). While there was no significant difference in median DTG time between direct-to-hub patients and non-telestroke transfers (83 vs 78 min, *P* = 0.75), DTG <60 min was achieved in a significantly higher proportion of non-telestroke transfer patients compared to direct-to-hub (38 vs 17%, *P* = 0.003) (Table 1).

In 53% of cases, CTA was performed at the spoke site prior to transfer to the hub institution. Further, 59% of telestroke transfer cases received a CTA prior to transfer compared to only 40% of non-telestroke transfers (59 vs 40%, *P* = 0.011) (Table 2). In all of the transfer patients who did not receive a CTA at the spoke, CTA was obtained upon arrival to the hub. As expected, median DTG time was shorter in transfer patients who received pre-transfer CTA compared to transfer patients who received CTA upon hub arrival (45 vs 83 min, *P* < 0.001) (Table 2). Further, there was no difference in median DTG time between transfer patients who received CTA upon hub arrival and patients who presented directly to the hub institution (direct-to-hub) (83 vs 83 min, *P* = 0.92). Similarly, DTG <60 min was achieved in 67% of transfer patients who received pre-transfer CTA compared to only 22% of transfer patients who received CTA upon hub arrival and 17% of patients who presented direct-to-hub (Table 2).

A repeat CTA was obtained upon arrival in 67% of transfer patients despite also having received a CTA at the spoke. There was no significant difference in the frequency of repeat CTA in telestroke compared to non-telestroke transfers (63 vs 86%, *P* = 0.09). As expected, in transfer patients who received pre-transfer CTA, median DTG was significantly shorter in patients who did not receive repeat CTA upon arrival at the hub compared to those with repeat CTA (26 vs 59 min, *P* < 0.001). Similarly, DTG <60 min was achieved in 100% of transfer patients who received pre-transfer CTA but did not require repeat CTA upon arrival at the hub compared to 53% who received pre-transfer CTA plus repeat at the hub (100 vs 53%, *P* < 0.001).

Interestingly, median DTG was shorter in patients who received pre-transfer CTA and subsequently received a repeat CTA upon hub arrival compared to transfer patients who only received a CTA upon arrival to the hub (59 vs 83, *P* = 0.02). Similarly, DTG <60 min was achieved in 53% of patients who received pre-transfer CTA and subsequently received a repeat CTA upon hub arrival compared to only 22% of transfer patients who only received a CTA upon arrival to hub (53 vs 22%, *P* < 0.001).

We used binary logistic regression analysis to determine which study variables could predict a DTG <60 min in transfer patients, controlling for the following variables entered in the first step: baseline NIHSS score; telestroke vs non-telestroke; CTA prior to transfer; and repeat CTA at the hub. The only variable that remained in the model was CTA prior to transfer, as transfer patients who received CTA prior to transfer were 7.2 times more likely to have a DTG in < 60 min compared to those who did not (odds ratio [OR] 7.2, 95% confidence interval [CI] 3.5–14.7; *P* < 0.001).

Despite the reduced DTG times among those with pre-transfer CTA, we observed no difference in patient outcomes on the basis of successful recanalization rate, length of stay, functional status (mRS), or mortality.

## DISCUSSION

Within our study population, pre-transfer CTA was the only significant predictor of achieving benchmark DTG metrics. Additionally, patients who received telestroke consultation were more likely to have received CTA prior to arrival. Given that a significant proportion of LVO-AIS patients will occur among patients far from EVT-capable centers, flexible solutions and innovation in practice are necessary to expedite treatment. One key component to the triage and transfer process is CTA acquisition.

Debate persists regarding whether pre-transfer CTA optimizes treatment or simply prolongs transfer to an EVT-capable center where image acquisition may be more operational. In their analysis of two telestroke networks, Al Kasab et al determined that AIS patients with LVO who received pre-transfer CTA had longer DTG times than those who received CTA at the hub hospital.^7^ This was attributed to the interventional team being activated at the hub hospital only after CTA was repeated by the direct-to-hub team; however, frequency of repeat CTA was not reported. In contrast, our study demonstrated that patients were 7.2 times more likely to have a DTG time <60 min if they underwent pre-transfer CTA.

Repeat CT head is obtained in patients with prolonged transfer time (>2 hours), and repeat CTA is typically performed in cases with lower Alberta Stroke Program Early CT Scores (ASPECTS), implying larger infarction core. Among our population, interfacility transport is vulnerable to a variety of factors including rural geography, a statewide lack of emergency medical services resources via ground or air, as well as weather patterns impeding helicopter transfer approximately 50% of the time. Given these factors, most transferred patients have prolonged transfer times, as demonstrated with a 67% rate of repeat imaging. A DTG <60 min was achieved in 100% of transfer patients who received pre-transfer CTA but did not require repeat CTA upon arrival at the hub, compared to 53% of patients who received pre-transfer CTA plus repeat at the hub (100 vs. 53%, *P* < 0.001). However, DTG <60 min was achieved in 53% of patients who received pre-transfer CTA and subsequently received a repeat CTA upon hub arrival compared to only 22% of transfer patients who only received a CTA upon arrival to hub (53 vs 22%, *P* < 0.001). Therefore, even in cases where CTA must be repeated at the hub, there is still a reduction in DTG time, and even including patients with repeat CTA, patients who received CTA prior to transfer were 7.2 times more likely to have a DTG time <60 min compared to those who did not (OR 7.2, 95% CI 3.5–14.7; *P* < 0.001). Therefore, the observed improvement in DTG time in transfer patients cannot be solely attributed to simply time saved by obtaining CTA prior to transfer; rather, it is likely related to advanced pre-notification and activation of the neurointerventional team, leading to more efficient triage and expedited ED course.

These findings underscore the value of enhanced pre-notification of incoming LVO patients. Pre-notification systems are established as effective ways to expedite treatment in time-critical conditions such as stroke.^12^–^14^ Telestroke facilitates streamlining of communication from spoke to hub, especially during the transfer process, and involvement of all stakeholders (receiving emergency medicine, neurology, and neuro-interventional teams) prior to patient transfer maximizes efficiency. Although implementation and maintenance of telestroke systems require significant resources and commitment from all network partners,^15^,^16^ the benefits from a patient and payor perspective are well-established.^17^–^21^ We have shown here that transfer patients from telestroke spoke sites were more likely to receive pre-transfer CTA than transfer patients from non-telestroke sites, with pre-transfer leading to improved DTG times. Sun et al demonstrated that DTG time, along with unmodifiable factors such as patient age, NIHSS score, and reperfusion status, was most strongly associated with favorable patient outcomes following AIS.^5^

While establishing or participating in a telestroke network may not be feasible in some areas, standardizing imaging protocols for all potential AIS-LVO patients should be a priority. The importance of an efficient triage process has been further highlighted by the COVID-19 pandemic. Disruptions in EVT-workflow became more common during the pandemic due to reallocation of staff.^10^ A more uniform triage process would limit delays in facilitating time-critical acute stroke therapy. Although telestroke consultation did not independently predict a shorter DTG time, pre-transfer CTA was obtained in a significantly higher proportion of telestroke compared to non-telestroke transfers, which may indirectly contribute to decreased DTG time.

Our findings demonstrate that patients who receive pre-transfer CTA are more likely to achieve DTG time ≤60 minutes. Including pre-transfer CTA as part of a standardized protocol for AIS patients could potentially decrease the time and resource burden on the over-populated and understaffed hub hospitals. Not only can CTA confirm that a patient has a target lesion amenable to EVT, but it equally serves to identify patients who are not candidates for intervention, thereby reducing futile transfers or avoiding resource-heavy helicopter flights. Although we did not observe improved outcomes among cases with shortened DTG times, it is well established that DTG time is a powerful predictor of clinical outcome.^5^,^6^,^22^

## LIMITATIONS

Our study has several limitations. First, the elapsed time from arrival to spoke to transfer from spoke—door in/door out time—could not be reliably obtained for all transfer patients. Therefore, we were unable to determine the net effect of pre-transfer CTA on door in/door out and DTG time. Future studies are necessary to evaluate door in/door out times at the transfer site, with and without CTA, to confirm that pre-transfer CTA does not delay time of transfer that exceeds the improvement of DTG time at the hub. Furthermore, while this study only evaluated DTG time at a single hub site, the diversity of geography (spanning four states) and volume (ranging from critical access hospitals to primary stroke centers) among participating spoke sites is also a notable strength. This data demonstrates that pre-transfer CTA can be reasonably acquired across a variety of practice environments.

We believe the findings of this study are generalizable to both rural and non-rural areas. Our rural academic center, which serves much of the 1.8-million-person population of West Virginia, is the only center with EVT-capabilities in the region. While many of our patients may be affected by factors that are more unique to this area, such as prolonged transportation times due to geography, nearly 50% of US residents live in areas with similar healthcare densities,^2^ indicating that this is a significant problem throughout the country. Thus, the association between obtaining pre-transfer vascular imaging and reduced DTG time is generalizable to both rural and non-rural areas.

Lastly, we observed no significant association between reduced DTG time and improved patient outcomes on the basis of successful recanalization rate, length of stay, functional status (mRS), or mortality. The absence of a clear outcome correlate in our data may be due to the relatively small sample size, as well as to our inability to control for all confounding factors, including individual site operating procedures, telestroke utilization rates, transfer modalities, and distance traveled to arrive at the hub.

## CONCLUSION

Reduction in door-to-groin time remains an important benchmark to advance the treatment of acute ischemic stroke patients with large vessel occlusions. Although several multitier, lean quality improvement initiatives have been developed to optimize workflow,^23^–^27^ our study provides evidence that focusing efforts on pre-transfer vascular imaging alone yields powerful results. Furthermore, our findings underscore an important implementation opportunity for emergency medicine leaders that also facilitates collaboration across disciplines and health systems.

## Figures and Tables

**Table 1:** Unadjusted comparison of direct-to-hub, telestroke transfer, and non-telestroke transfer patients.

Variable	Direct to hub (n = 189)	Telestroke Transfer (n = 134)	Non-telestroke Transfer (n = 60)	*P*-value
Baseline NIHSS (mean ± SD)	14 ±6	14 ±7	15 ± 8	0.478
Door-to-groin time (DTG) (median [IQR] minutes)	83 [37]	61 [47]	78 [50]	<**0.0001**
DTG time <60 min (%)	17	50	38	<**0.0001**
EVT recanalization (%)	37	42	30	0.329
Length of stay (median [IQR] days)	5 [4]	7 [7]	7 [10]	0.108
Discharge mortality (%)	17	19	19	0.832
90-day mRS 0–2 (%)	36	24	32	0.081

*NIHSS*, National Institutes of Health Stroke Scale*; EVT*, endovascular therapy; *IQR*, interquartile range; *mRS*, modified Rankin Scale.

**Table 2:** Unadjusted comparison of transfer patients by computed tomography angiography status.

Variable	CTA obtained prior to transfer (n = 102)	CTA upon hub arrival only (n = 92)	*P*-value
Received telestroke consultation (%)	59	40	**0.011**
Door-to-groin time (DTG) (median [IQR] minutes)	45 [46]	83 [47]	<**0.001**
DTG <60 min (%)	67	22	<**0.001**
Repeat CTA at hub (%)	67	-	**-**

*CTA*, computed tomography angiography; *IQR*, interquartile range.
